# Detection of Toxin Genes and Biofilm Formation of *Pseudomonas aeruginosa* Associated With Respiratory Tract Infections in Riyadh, Saudi Arabia

**DOI:** 10.1155/ijm/1302634

**Published:** 2026-04-13

**Authors:** Reem Aljaaidi, Maryam Alshammari, Manal AlKhulaifi, Bader Alrashidi, Abdulkarim Alhetheel, Enshad Alzaidi, Dunia Al Farraj

**Affiliations:** ^1^ Department of Botany and Microbiology, King Saud University, Riyadh, Saudi Arabia, ksu.edu.sa; ^2^ College of Applied Medical Sciences, Inaya Medical Colleges, Riyadh, Saudi Arabia; ^3^ Department of Pathology, College of Medicine, King Saud University, Riyadh, Saudi Arabia, ksu.edu.sa

**Keywords:** antibiotic resistance, biofilm formation, exoenzyme S, exotoxin A, *Pseudomonas aeruginosa*, respiratory tract infections

## Abstract

*Pseudomonas aeruginosa* infections associated with the respiratory system may cause significant damage to the lung tissue, which may lead to death. This study aimed to detect the presence of toxin genes, antibiotic resistance, and biofilm formation in *P. aeruginosa* isolates associated with respiratory tract infections. A total of 60 *P. aeruginosa* strains were isolated from respiratory samples and included in the current study after confirmation using biochemical identification methods. Antimicrobial susceptibility was conducted using the MicroScan WalkAway system. Biofilm formation was assessed using the 96‐microtiter plate method. Conventional polymerase chain reaction (PCR) was used to detect the *tox*A and *exo*S genes. The results showed that 25% of *P. aeruginosa* isolated from respiratory tract infections were resistant to Imipenem, while most isolates were sensitive to colistin (98.3%). Among the isolates, 96.6% were biofilm producers as follows: 55% were strong biofilm producers, 18.3% were moderate biofilm producers, and 23.3% were weak biofilm producers, while 3.3% of the isolates did not produce biofilms. The *tox*A gene was present in 93.3% of isolates, and the *exo*S gene was present in 61.7%. However, our finding revealed that there is no significant correlation between the presence of toxin genes and biofilm formation as well as antibiotic resistance among the isolates. The current study assumes that each of the mentioned virulence factors may be used by *P. aeruginosa* during a certain stage of infection to perform a specific role. In conclusion, the presence of virulence factors such as toxin genes, biofilm formation, and antibiotic resistance in *P. aeruginosa* may exacerbate respiratory tract infections.

## 1. Introduction

*Pseudomonas aeruginosa* is a Gram‐negative pathogen causing acute or chronic infection in immunocompromised persons with chronic obstructive pulmonary disease, cancer, cystic fibrosis, sepsis, burns, and ventilator‐associated pneumonia [1]. Chronic respiratory infections caused by *P. aeruginosa* lead to inflammation and lung tissue damage, with the produced exopolysaccharide (EPS) covering the airways [2]. A significant exacerbation of these infections can result in increased mortality among chronic respiratory patients. [2]. In addition, *P. aeruginosa* exhibits resistance to many disinfectants and multiple classes of antibiotics. Its prevalence is particularly high in hospital environments, especially in intensive care units [3]. This organism exhibits intrinsic and acquired resistance to a variety of antimicrobial classes [4].

Antibiotic resistance among *P. aeruginosa* strains has become a major global public health concern [5, 6]. Owing to the presence of diverse resistance mechanisms, many clinical isolates show reduced susceptibility to commonly used antibiotics [2]. Moreover, *P. aeruginosa* is known for its rapid mutation and adaptation to achieve antibiotic resistance, making the infection very challenging to treat [7]. The effective therapeutic options for multidrug‐resistant (MDR) *P. aeruginosa* infections remain scarce [8]. Global reports indicated a significant increase in the incidence of MDR and XDR among *P. aeruginosa* strains in hospitalized patients [6, 9, 10].

*P. aeruginosa* forms membrane‐bound biofilms that serve as an important defense mechanism [11]. Bacterial biofilms are communities composed of various bacteria adhered to a biotic or abiotic surface and surrounded by an EPS matrix to protect the microorganisms from external or internal microbial aggressions [12]. The ability to form biofilms is typically associated with bacterial survival, enhanced growth, increased resistance to antibiotic therapy, and the ability to withstand hazardous environmental conditions [13, 14]. Biofilms are therapeutically significant due to their decreased antibiotic susceptibility, which serves as a population strategy for bacterial persistence under stress. Bacterial cells communicate through quorum sensing, a regulatory system that controls gene expression in response to changes in cell density. In *P. aeruginosa*, biofilm formation is primarily regulated by quorum sensing [15, 16].

**FIGURE 1:**
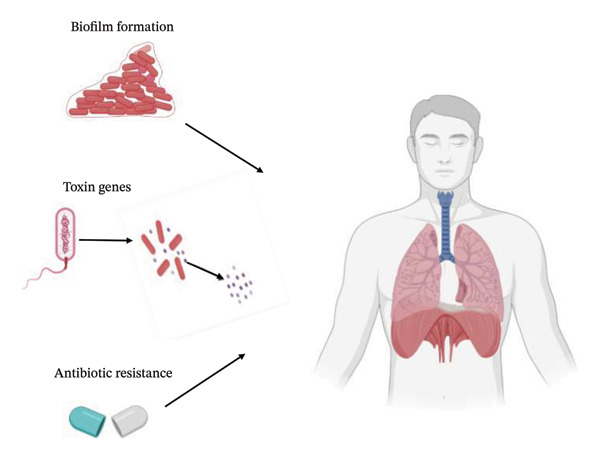
Schematic representation of a proposed approach to detect the relation between toxin genes, antibiotic resistance, and biofilm formation in *P. aeruginosa* associated with respiratory tract infections. The figure was created with BioRender (https://biorender.com/).

Additionally*, P. aeruginosa* secretes several extracellular virulence factors, mainly toxins such as toxin A, exotoxin T, and exoenzyme S, which play a crucial role in its pathogenicity [17]. These virulence factors enhance colonization, adhesion, biofilm formation, and tissue damage, contributing to the development of chronic pneumonia [18]. The primary lethal virulence factor of *P. aeruginosa* is exotoxin A, which is produced by most strains and encoded by the *tox*A gene. It has ADP‐ribosylation activity, which affects the protein synthesis of the host cell, has a necrotizing effect on tissues, and facilitates bacterial colonization [19, 20]. Exoenzyme S (*exo*S) is a bifunctional ADP ribosyltransferase (ADPRT) and GTPase‐activating protein (GAP). It plays a critical role in bacterial invasion, colonization, and dissemination and also inhibits phagocytosis in pneumonia [21, 22]. Thus, the main purpose of this study is to detect the presence of the toxin genes, antibiotic resistance, and biofilm formation in *P. aeruginosa* associated with respiratory tract infections, in addition to analyzing the potential relationships among these virulence factors (Figure 1).

## 2. Materials and Methods

### 2.1. Samples Collection, Isolation, and Identification

A total of 60 *P. aeruginosa* were isolated from respiratory samples of patients with respiratory tract infections, including both children and adults, at King Saud University Medical City (KSUMC) in Riyadh. Phenotypic and biochemical characterization of the *P. aeruginosa* isolates was performed. *P. aeruginosa* isolates were identified using the MicroScan WalkAway system (Siemens, Tarrytown, NY).

### 2.2. Antimicrobial Susceptibility Testing

An antimicrobial susceptibility test was performed using the MicroScan WalkAway system (Siemens, Tarrytown, NY). *P. aeruginosa* ATCC 27853 was used as a positive control. The MIC values were interpreted according to Clinical and Laboratory Standards Institute guidelines (CLSI) [23]. The system categorized antimicrobial susceptibility results as resistant (R), intermediate (I), and sensitive (S). Isolates that showed resistance to at least one antibiotic from three or more different classes were considered as MDR [24].

### 2.3. Biofilm Formation Assay

To examine biofilm formation, freshly prepared *P. aeruginosa* cultures were inoculated into 3 mL of Luria‐Bertani broth (Lb broth) medium. The bacterial suspension was adjusted according to the 0.5 McFarland standards. The 96‐well microtiter plate was then filled with 200 μL of the bacterial suspensions. Each strain was tested three times in three independent experiments. The plates were incubated at 37°C for 24°h without shaking. After that, the plates were washed three times with double‐distilled water, the wells were stained with 225 μL of 1% crystal violet for 10 min, and then the plates were washed and dried for 10 min at room temperature. Subsequently, 225 μL of acetic acid was added to each well and incubated at room temperature for 10 min. Then, crystal violet was solubilized and cell density was quantified using a BioTek microplate reader at 560 nm (OD560) (Synergy 2, BioTek). *P. aeruginosa* PAO1 was used as a positive control, and the Lb broth only was used as a negative control. The isolated *P. aeruginosa* strains were categorized into four classes based on their adherence capabilities: non–biofilm producer, weak biofilm producer, moderate biofilm producer, and strong biofilm producer (Table 1) [25].

**TABLE 1:** Biofilm formation categories.

Biofilm formation capacity	Mean of optical density
Negative	OD ≤ ODc
Weak	2 × ODc ≥ OD > ODc
Moderate	4 × ODc ≥ OD > 2 × ODc
Strong	OD > 4 × ODc

*Note:* ODc = average OD of negative control + (standard deviations [SD] of negative control × 3).

Abbreviation: ODc, optical density cutoff.

### 2.4. Detection of *tox*A and *exo*S in *P. aeruginosa* Isolates

All *P. aeruginosa* isolates were screened for the presence of the *tox*A and *exo*S genes using suitable primers as described in Table 2 [26]. DNA was automatically extracted using Qiagen EZ1 Advanced XL according to the manufacturer’s instructions (Qiagen, EZ1 Virus Mini Kit v2.0). Following DNA extraction, polymerase chain reactions (PCRs) were performed using the Applied Biosystems GeneAmp PCR System 9700 to detect the *tox*A and *exo*S genes. The PCR was performed in a reaction mixture with total volume of 20 μL, containing 1 μL of template DNA, 1 μL of each primer, 10 μL of 2x Taq Master Mix, and 7 μL of nuclease‐free water. PCR conditions for *tox*A were as follows: DNA denaturing for 2 min at 95°C and amplifying for 35 cycles (1 min at 94°C, 1 min at 59°C, 1 min at 72°C for denaturation, annealing, and extension phases, respectively), followed by an additional period of extension for 10 min at 72°C. Similarly, for *exo*S except for 35 cycles (1 min at 94°C, 30 s at 57°C, 30 s at 72°C for denaturation, annealing, and extension phases). The amplified DNA was separated by electrophoresis on a 1.5% (w/v) agarose gel, loaded with 7.5 μL of DyeAll Dye. About 10 μL of sample was loaded into the electrophoresis system at 120 V for 75 min. The amplified bands were visualized and photographed under UV light using the WiseDoc System.

**TABLE 2:** Primer sequences used for the detection of *P. aeruginosa tox*A and *exo*S toxin genes.

Gene	Forward primer	Reverse primer	Size (bp)
*tox*A	GAC​AAC​GCC​CTC​AGC​ATC​ACC​AGC	CGC​TGG​CCC​ATT​CGC​TCC​AGC​GCT	396
*exo*S	GCG​AGG​TCA​GCA​GAG​TAT​CG	TTC​GGC​GTC​ACT​GTG​GAT​GC	118

### 2.5. Statistical Analysis

All collected data were analyzed using version 22.0 of Statistical Package for Social Sciences (SPSS) (SPSS Inc, Armonk, New York, USA). The Chi‐square test was used to compare the biofilm formation, toxin genes, and antibiotic susceptibility of *P. aeruginosa* isolated from respiratory tract samples. The overall level of statistical significance was set at *p* < 0.05.

## 3. Results

### 3.1. Bacterial Isolates and Patient Distribution

A total of 60 *P. aeruginosa* isolates were used in this study. The majority of the isolates were obtained from sputum samples, accounting for 73.3% (44/60) of the total isolates (Table 3). Overall, 55% (33/60) of *P. aeruginosa* strains were isolated from males, while 45% (27/60) were from females. Patients were categorized into three age groups: children (< 18 years), adults (18–59 years), and older adults (≥ 60 years). The pediatric group was further subdivided into two categories: < 5 years and 5–17 years (Table 4).

**TABLE 3:** Sources of respiratory isolates for *P. aeruginosa*.

Sources	Number	%
Sputum	44	73.3
Induced sputum	2	3.3
Endotracheal asp	3	5.0
Tracheal asp	8	13.3
BAL	2	3.3
Bronchial wash	1	1.7
Total	60	

*Note:* BAL: Bronchoalveolar Lavage.

Abbreviations: endotracheal Asp, endotracheal aspirate; tracheal asp, tracheal aspirate.

**TABLE 4:** *P. aeruginosa* isolates according to gender and age.

Gender	Age group (%)				Total
	≤ 5	6–17	18–59	≥ 60	
Female	3 (5.0)	5 (8.3)	12 (20.0)	7 (11.7)	27 (45.0)
Male	2 (3.3)	3 (5.0)	19 (31.7)	9 (15.0)	33 (55.0)
Total	5 (8.3)	8 (13.3)	31 (51.7)	16 (26.7)	60

### 3.2. Characteristics of *P. aeruginosa* Isolates

The bacterial isolates showed the morphological and biochemical properties of *P. aeruginosa*. They were Gram‐negative, rod‐shaped, green‐yellow colonies on MacConkey agar and were oxidase‐positive. The colonies were smooth, the edges were flat and elevated, and some isolates also showed mucoid colonies.

### 3.3. Determination of Antimicrobial Susceptibility of *P. aeruginosa* Isolates

The results of the susceptibility testing of 60 *P. aeruginosa* isolates were categorized as sensitive, intermediate, and resistant, as shown in Table 5. The highest resistance rate was observed against Imipenem and meropenem 26.7% (16/60) followed by Aztreonam 25% (15/60). It was also found that most of the isolates were sensitive to colistin 98.3% (1/60). A total of 25% (15/60) isolates were MDR, and 75% (45/60) were non‐MDR.

**TABLE 5:** Antibiotics susceptibility profiles and their relationship to the presence of toxin genes and biofilm formation in *P. aeruginosa* isolates.

Antibiotic	(+) *toxA* (*n* = 56) R (%)	*p* value	(+) *exo*S (*n* = 37) R (%)	*p* value	(+) BF (*n* = 58) R (%)	*p* value	Total R (*n* = 60) (%)
TZP	12 (21.4)	0.443	7 (18.9)	0.887	12 (20.7)	0.825	12 (20.0)
MEM	13 (23.2)	0.111	6 (16.2)	0.231	16 (27.6)	0.281	16 (26.7)
IMP	13 (23.2)	0.111	6 (16.2)	0.231	16 (27.6)	0.281	16 (26.7)
CAZ	13 (23.2)	0.704	7 (18.9)	0.638	13 (22.4)	0.584	13 (21.7)
FEP	11 (19.6)	0.490	6 (16.2)	0.730	11 (19.0)	0.887	11 (18.3)
AZT	14 (25.0)	0.748	9 (24.3)	0.931	15 (25.9)	0.354	15 (25.0)
GM	12 (21.4)	0.443	5 (13.5)	0.198	12 (20.7)	0.825	12 (20.0)
TOB	4 (7.1)	0.354	1 (2.7)	0.126	5 (8.6)	0.815	5 (8.3)
AK	7 (12.5)	0.490	4 (10.8)	0.710	8 (13.8)	0.704	8 (13.3)
CIP	7 (12.5)	0.088	4 (10.8)	0.222	9 (15.5)	0.156	10 (16.7)
LEV	4 (7.1)	0.211	3 (8.1)	0.718	5 (8.6)	0.584	6 (10.0)
COL	1 (1.8)	0.815	1 (2.7)	0.587	1 (1.7)	0.949	1 (1.7)

*Note:* (+), positive for toxin gene; AK, amikacin; AZT, aztreonam; BF, biofilm formation (weak, moderate, and strong); CAZ, ceftazidime; CIP, ciprofloxacin; COL, colistin; FEP, cefepime; GM, gentamicin; IPM, imipenem; LEV, levofloxacin; MEM, meropenem; R, resistant and intermediate; TOB, tobramycin; TZP, piperacillin/tazobactam.

### 3.4. Assessment of Biofilm Formation in *P. aeruginosa* Isolates

The biofilm‐forming ability was assessed for all 60 *P. aeruginosa* isolates using 96‐microtiter plate method. Results revealed that 96.6% of the isolates were positive biofilm producers. Only 3.3% of the isolates were non–biofilm producers. Despite the high prevalence of biofilm production, no statistically significant association was found between biofilm formation ability and MDR (*p* = 0.152) (Table 6).

**TABLE 6:** Degrees of biofilm formation among *P. aeruginosa* strains.

Degree of biofilm formation	No. of isolates (%)
Strong^d^	33 (55)
Moderate^c^	11 (18.3)
Weak^b^	14 (23.3)
None^a^	2 (3.3)
Total	60

*Note:* Biofilm‐forming ability was classified as weak, moderate, and strong. The negative control was defined by a cutoff (ODc = 0.08).

^a^OD ≤ ODc (0.08).

^b^2 × ODc (0.164) ≥ OD > ODc.

^c^4 × ODc (0.328) ≥ OD > 2 × ODc (0.164).

^d^OD > 4 × ODc (0.328).

### 3.5. Detection of Toxin Genes (*tox*A and *exo*S) of *P. aeruginosa*

**FIGURE 2:**
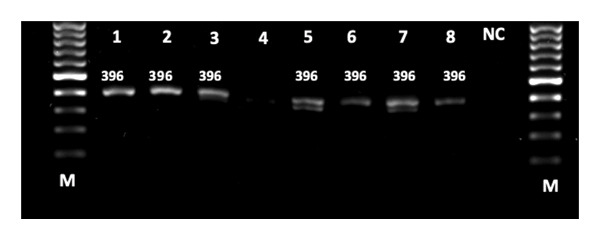
Detection of amplified *tox*A gene from *P. aeruginosa* strains using agarose gel electrophoresis. Lanes 1–8 = samples; NC = negative control; M = 100 bp DNA marker; MW = 396 bp. Note. MW = molecular weight; bp = base pair.

**FIGURE 3:**
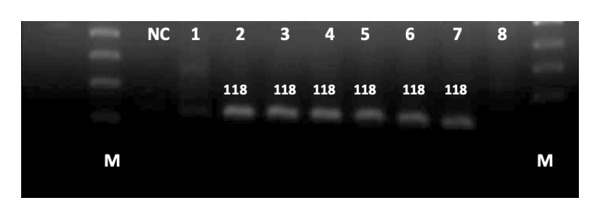
Detection of amplified *exo*S gene from *P. aeruginosa* strains using agarose gel electrophoresis. NC = negative control; lanes 1–8 = samples; M = 100 bp DNA marker; MW = 118 bp.

Among 60 strains, 93.3% *P. aeruginosa* isolates possessed the *tox*A gene, and 61.7% contained the *exo*S gene. *tox*A is the most prevalent gene among the isolates. No association was observed between the presence of toxins genes and biofilm formation as well as antibiotic resistance (Table 7) (Figure 2) (Figure 3).

**TABLE 7:** Prevalence of toxin genes and their association with biofilm formation and multidrug resistance among *P. aeruginosa* isolates.

Toxin genes	Degree of biofilm formation				*p* value	Prevalence of multidrug resistance		*p* value
	Strong	Moderate	Weak	None		MDR	Non‐MDR	
*tox*A+	29 (48.33%)	11 (18.33%)	14 (23.33%)	2 (3.33%)	0. 320	13 (21.66%)	43 (71.66%)	0. 232
*tox*A–	4 (6.66%)	0 (0%)	0 (0%)	0 (0%)		2 (3.33%)	2 (3.33%)	
*eox*S+	16 (26.66%)	9 (15%)	10 (16.66%)	2 (3.33%)	0. 106	8 (13.33%)	29 (48.33%)	0. 443
*eox*S–	17 (28.33%)	2 (3.33%)	4 (6.66%)	0 (0%)		7 (11.66%)	16 (26.66%)	

## 4. Discussion

The present work aimed to study the presence of the toxin genes and biofilm formation and antibiotic resistance in *P. aeruginosa* that is associated with respiratory tract infection. The present study revealed that *P. aeruginosa* was one of the most common bacterial isolates from respiratory samples. This result is consistent with a previous study conducted in Saudi Arabia, which reported *Acinetobacter* spp. (27.2%) followed by *P. aeruginosa* (23.8%) as predominant respiratory pathogens [27]. In similar studies, the prevalence of 32.1% and 20.3% of *P. aeruginosa* was reported in Gujarat, India [28, 29]. In comparison, a higher prevalence rate of *P. aeruginosa* isolates (66.7%) was obtained from respiratory samples [30]. This indicates the prevalence of *P. aeruginosa* in samples associated with respiratory tract infections. The prevalence of *P. aeruginosa* was higher in males (55%) than in female patients. Similar observations were made previously by Siddiqua et al. (75.36%) and Andhale et al. (76.66%). Male predominance may be attributed to personal habits, smoking, outdoor activities, work‐related factors, and exposure to water, soil, and other environments inhabited by the organism [31, 32]. In this study, *P. aeruginosa* strains were isolated from most of the patients who belonged to the adult age group (18–59 years) 31 (52%), and the elderly age groups of ≥ 60 years 16 (27%). This may be attributed to prolonged hospitalization, reduced immunity, and other comorbidities [32]. The observed predominance of *P. aeruginosa* isolates appears to be multifactorial, potentially driven by the synergistic effect of its intrinsic resistance mechanisms and robust biofilm‐forming capacity. These attributes not only facilitate environmental persistence but also contribute significantly to the evasion of host immune responses and subsequent therapeutic failure.

The drug‐resistance patterns may differ in each country and region as a result of genetic alteration mediated by inappropriate antibiotic consumption or usage [33]. Our finding revealed that the majority of *P. aeruginosa* isolates were sensitive to Colistin (98.3%), followed by Tobramycin (91.7%). However, increased resistance to imipenem and meropenem (26.7%) and aztreonam (25%) indicates a gradual decline in carbapenem efficacy. Similar resistance trends have been reported in Egypt, with higher resistance rates to tobramycin (46%) and imipenem (38%) [34]. In contrast, other studies documented differing patterns, including higher resistance to amikacin (48%) and lower resistance to imipenem (7%) [35], highlighting pronounced regional variability. The frequency of MDR *Pseudomonas* in this study was 25%. In a recent retrospective study in Saudi Arabia, respiratory samples were the most common source of MDR (58.9%) [36]. Additionally, according to a recent national report by Centers for Disease Control and Prevention (CDC), hospital‐acquired MDR *P. aeruginosa* infections increased by 35% between 2019 and 2020 [37]. This rise is attributed to the increased usage of antibiotics at that time to treat secondary bacterial infections associated with SARS‐CoV‐2 infection [36]. The increasing threat of antimicrobial resistance in *P. aeruginosa* is because it has the extraordinary ability to develop resistance to almost all available antibiotics due to its ability to undergo mutations in chromosomal genes and the increasing prevalence of transferable resistance determinants [38].

The formation of biofilms by *P. aeruginosa* represents a critical virulence strategy that significantly enhances its pathogenesis by creating a physical and metabolic shield against antimicrobial penetration [39]. In this study, a vast majority of *P. aeruginosa* isolates (96.6%) were positive for biofilm producers, with more than half (55%) classified as strong producers. These findings are relatively consistent with reports showing high biofilm prevalence, including El‐Khashaab et al. (91.4%), and similar rates were documented in other studies (86.5% and 83.75%) [40–42]. Conversely, these results contrast sharply with the much lower rate (27%) reported by Abdelraheem et al. [35]. In fact, biofilm‐forming bacteria can increase resistance to antibiotics. This antibiotic resistance is due to reduced antibiotic diffusion through the EPS matrix of biofilms, and the production of specific antibiotic resistance factors [43, 44]. Although all MDR isolates in the current study were biofilm producers, this association was not statistically significant. This finding is consistent with Corehtash et al., who reported MDR isolates in both biofilm‐positive and biofilm‐negative groups, with the majority significantly associated with biofilm formation [45]. Three hypotheses are proposed for how biofilms are resistant to antibiotics: The expulsion of antibiotics from the biofilm due to cumulative “efflux action” by the microbial populations, antibiotics’ inability to penetrate the thick matrix, and because most of the pathogens in the deeper layers of the biofilm are metabolically inactive, the antibiotic is unable to inhibit pathogens [46]. Biofilms increase the complexity of treatment options, therefore requiring a new therapeutic strategy that combines conventional antibiotics with a substance that interferes with biofilms, rendering them more susceptible to treatment [47].

Extracellular virulence factors, such as toxins encoded by the *tox*A and *exo*S genes contribute to bacterial dissemination, host invasion, and extensive tissue damage in *P. aeruginosa* infections [48]. In the present study, a high prevalence of *tox*A (93.3%) and a moderate prevalence of *exo*S (61.7%) were detected among *P. aeruginosa* isolates, highlighting the substantial role of toxin gene–mediated mechanisms in the pathogenesis of respiratory tract infections. The high frequency of *tox*A observed in this study is consistent with reports by Chand et al. (95.4%) and studies from Iran (69.4%), supporting the notion that *tox*A is widely conserved among clinical *P. aeruginosa* isolates [49, 50]. In contrast, the prevalence of *exo*S exhibited greater variability across studies, with lower rates reported previously (45.4%) and substantially higher rates documented in Nigeria, where all *P. aeruginosa* isolates were positive for both *tox*A and *exo*S genes (100%) [51, 52]. The divergences in the prevalence of virulence genes in *P. aeruginosa* may depend on several different factors, including the immune status of individual patients, the virulence of the strains, type and degree of contamination, and environmental sources [53]. Although both *tox*A and *exo*S genes contribute to pathogenicity and antimicrobial resistance in *P. aeruginosa* isolates from respiratory samples, *tox*A was the predominant gene among MDR *P. aeruginosa* isolates. However, no statistically significant association was observed between *tox*A carriage and MDR status (*p* = 0.232). In contrast, Khosravi et al. (2016) reported a statistically significant difference (*p* < 0.001) in the prevalence of *tox*A and *exo*S genes between MDR and non‐MDR strains [20]. The coexistence of toxin genes and biofilm‐forming capacity in *P. aeruginosa* may confer a selective advantage by enhancing colonization, antimicrobial resistance, and persistence of pathogenicity in respiratory tract infections.

The present findings may help establish appropriate anti‐virulence and anti‐biofilm programs as adjuncts to conventional therapies to control respiratory infections and reduce biofilm‐mediated drug resistance in MDR *P. aeruginosa*.

### 4.1. Limitations

A primary limitation of this study is the lack of access to patient clinical histories, such as other illnesses or previous antibiotic treatments, and ward distribution, as administrative and ethical approvals for accessing individual medical records were not obtained.

## 5. Conclusion

To our knowledge, this study represents one of the few reports from Riyadh, Saudi Arabia, investigating the phenotypic and genotypic characteristics of *P. aeruginosa* associated with respiratory tract infections, with emphasis on toxin genes, biofilm formation, and antimicrobial resistance. The findings demonstrated a high prevalence of toxin genes, particularly *tox*A (93.3%), while 96.6% of *P. aeruginosa* isolates exhibited biofilm‐forming ability. Although no significant association was observed between toxin gene and MDR, the coexistence of toxin genes and biofilm formation may contribute to bacterial persistence and reduced antimicrobial efficacy. Further studies are recommended to investigate the meticulous correlation between toxin genes, with biofilm formation and antimicrobial resistance in *P. aeruginosa* to support the development of effective therapeutic and infection control strategies.

## Funding

This study was financially supported by Ongoing Research funding program, King Saud University, Riyadh, Saudi Arabia.

## Ethics Statement

The authors have nothing to report.

## Conflicts of Interest

The authors declare no conflicts of interest.

## Data Availability

Data sharing is not applicable to this article as no datasets were generated or analyzed during the current study.
